# Total Glucosides of Danggui Buxue Tang Attenuate BLM-Induced Pulmonary Fibrosis via Regulating Oxidative Stress by Inhibiting NOX4

**DOI:** 10.1155/2015/645814

**Published:** 2015-08-11

**Authors:** Ping Zhao, Wen-Cheng Zhou, De-Lin Li, Xiao-Ting Mo, Liang Xu, Liu-Cheng Li, Wen-Hui Cui, Jian Gao

**Affiliations:** ^1^School of Pharmacy, Anhui University of Chinese Medicine, Hefei 230038, China; ^2^The First Affiliated Hospital of Anhui Medical University, Hefei 230022, China; ^3^School of Pharmacy, Anhui Medical University, Hefei 230032, China

## Abstract

Pulmonary fibrosis (PF) is a serious chronic lung disease with unknown pathogenesis. Researches have confirmed that oxidative stress which is regulated by NADPH oxidase-4 (NOX4), a main source of reactive oxygen species (ROS), is an important molecular mechanism underlying PF. Previous studies showed that total glucosides of Danggui Buxue Tang (DBTG), an extract from a classical traditional Chinese herbal formula, Danggui Buxue Tang (DBT), attenuated bleomycin-induced PF in rats. However, the mechanisms of DBTG are still not clear. We hypothesize that DBTG attenuates PF through regulating the level of oxidative stress by inhibiting NOX4. And we found that fibrosis indexes hydroxyproline (HYP) and type I collagen (Col-I) were lower in DBTG groups compared with the model group. In addition, the expression of transforming growth factor-*β*1 (TGF-*β*1) and expression of alpha smooth muscle actin (*α*-SMA) were also much more decreased than the model group. For oxidative stress indicators, DBTG blunted the decrease of superoxide dismutase (SOD) activity, total antioxidant capacity (T-AOC), and the increase in malondialdehyde (MDA), 8-iso-prostaglandin in lung homogenates. Treatment with DBTG restrained the expression of NOX4 compared to the model group. Present study confirms that DBTG inhibits BLM-induced PF by modulating the level of oxidative stress via suppressing NOX4.

## 1. Introduction

Pulmonary fibrosis (PF) is a chronic lung disease and characterized by excessive accumulation of extracellular matrix (ECM) deposition to mesenchymal transition (EMT), which finally leads to the decline of lung function [1]. Because environmental pollution is more and more serious, the incidence of PF is on the rise in China recently [2]. Although the mechanisms of PF are not understood fully, transforming growth factor-*β*1 (TGF-*β*1) is recognized as a critical factor to induce fibrosis [3]. More and more researches have confirmed that oxidative stress is an important molecular mechanism underlying fibrosis in a variety of organs, especially for lung [4]. After being stimulated by dust, drugs, and other factors, oxidative stress usually occurred in lung and the balance between oxides and peroxides will be broken; then, the level of reactive oxygen species (ROS) will be increased because of oxidative stress. ROS induces PF by promoting apoptosis of alveolar epithelial cells (AECs), inflammatory cell infiltrating, accumulation of collagen, and ECM [5]. Oxidative stress is frequently defined as the imbalance of oxidant production and antioxidant defenses and leads to cellular dysfunction and tissue damage [6]. NOX4 belongs to the NADPH oxidase (NOX) family of enzymes, which catalyze the reduction of O_2_ to form ROS [7]. It is one of the most important sources of ROS in vivo. Researches have shown that NOX4 is closely associated with the development of PF comparing to other enzymes in NOX family [8].

Danggui Buxue Tang (DBT) composed of Radix Astragali (RA) and Radix Angelicae Sinensis (RAS) at a ratio of 5 : 1 is a classical traditional Chinese herbal formula [9]. DBT is traditionally used by women as a remedy for menopausal symptoms, in China [9]. Nowadays, recent pharmacological researches confirmed that DBT had the ability to improve haematopoietic function, stimulate cardiovascular circulation, prevent osteoporosis, increase antioxidation activity, and stimulate the immune system [10, 11]. Total glucosides of Danggui Buxue Tang (DBTG) is an extract from DBT. Our previous studies have confirmed that DBTG attenuated bleomycin- (BLM-) induced PF in rats via inhibition of ECM [12, 13]. Since the mechanisms of DBTG are still unclear, we hypothesize that DBTG attenuates PF through regulating the level of oxidative stress by inhibiting NOX4. The results of the present study may explore other potential mechanisms of DBTG and provide the basis for the clinical treatment of PF.

## 2. Materials and Methods

### 2.1. Animals

Sprague-Dawley rats weighing 180–220 g (*n* = 210) were obtained from the Laboratory Animal Department of Anhui Medical University, Hefei, China. Rats were housed at a constant room temperature (23 ± 2°C), humidity (60 ± 10%), and light cycle (12 : 12 h light-dark), with free access to tap water as well as standard chow. All rats' experimental procedures were approved by Anhui Medical University Animal Care Committee and followed the protocol outlined in The Guide for the Care and Use of Laboratory Animals published by the USA National Institute of Health (NIH Pub. No. 85–23, Revised 1996).

### 2.2. Preparation of DBTG and Content Determination

Two different medicinal herbs that consist of DBT were purchased from the pharmacy of The First Affiliated Hospital of Anhui Medical University, China, and all of them were identified by Professor Wang De-qun (Anhui College of Traditional Chinese Medicine, Hefei, China) as Astragali Radix [root of Astragalus membranaceus the (Fisch.) Bge. var. mongholicus (Bge.) Hsiao (Huangqi, 14021203)] and Angelicae Sinensis Radix [root of Angelicae Sinensis (Oliv.) Diels (Danggui, 13041502)]. The method of extraction DBTG was reported in our previous study [9, 10]. In brief, DBTG would be extracted by the following method.* Astragalus membranaceus* and Radix* Angelicae sinensis* were soaked in water for an hour and reflux was then heated three times (2 h per time). The extract would be filtered, enriched, and placed in 4°C for one night. The second day, the extract was centrifuged in 3000 r and impurities were removed. The supernatant was used to extract DBTG through macroporous silica gel D101. First water was used to remove the polysaccharide substance by slowly flushing silica gel column. Other impurities were discarded by 40% alcohol after water was flushed. The solution after 80% alcohol flushed was saved. DBTG would be got after drying the 80% alcohol solution. The content and fingerprint of DBTG were detected as the methods that previously described in Chinses [14, 15]. The average content of DBTG was 70.7%.

### 2.3. Experiment Design

Animals were randomly divided into the following seven groups (*n* = 30 per group): group 1 [control + no treatment], group 2 [sham-operated (sham) group received only NS + no treatment], group 3 [a dose of 5 mg·kg^−1^ bleomycin (BLM) induced PF model + no treatment], group 4/5/6 [a dose of 5 mg·kg^−1^ BLM induced PF model + a single oral dose of 4/8/16 mg·kg^−1^ DBTG treatment], and group 7 [a dose of 5 mg·kg^−1^ BLM induced PF model + a single oral dose of 5 mg·kg^−1^ prednisone treatment].

Bleomycin-A5 (Harbin Laiboten Pharmaceutical Co. LTD., Harbin, China) was dissolved by sterile normal saline (NS) with a volume of 1.6 mL before PF modeling. Animals in 3,4,5 and 6 group were treated with BLM at a dose of 5 mg·kg^−1^ by intratracheal instillation. And animals in group 2 were just treated with sterile NS in the same mode of administration. DBTG and prednisone (5 mg × 100 tablets, no. 1210246, Xinhua Pharmaceutical CO., LTD, Shandong, China) were dissolved by 0.5% CMC-Na the day before administration. The first day after modeling, 0.5% CMC-Na (1.2 mL·kg^−1^) was given to rats in groups 1, 2, and 3 by intragastric administration (ig) once daily. Groups 3, 5, and 6 were treated with DBTG (4/8/16 mg·kg^−1^, per day, ig) and rats received prednisone (5 mg·kg^−1^, per day, ig). Body weight of rats was recorded daily and animals were not sacrificed if they showed weight loss above a certain threshold. Rats were sacrificed with anaesthetizing by an intraperitoneal injection of pentobarbital (40 mg/kg) on days 7, 14, and 28 after modeling and then samples were obtained.

### 2.4. Sample Collection and Handling

Lung tissues were collected on days 7, 14, and 28 after modeling. Lung tissue was flushed with saline until no blood in a dish, and then the lung was weighed. Lung coefficient (lung weight (mg)/body weight (g)) would be calculated at each time point. Half of the left lung was fixed in 4% phosphate-buffered paraformaldehyde for histopathologic and immunohistochemistry preparation, while the other was frozen after using for homogenate. Lung tissues were homogenized in at 4°C saline three times with a polytron homogenizer (10 s of homogenization at 10 s intervals). And supernatant was reserved after homogenate centrifuged at 3000 g for 10 minutes and stored at −80°C for further analysis.

### 2.5. Histopathologic and Immunohistochemical

Lung samples were fixed in 10% phosphate-buffered formalin for 24 h. The median portion was cut and embedded in paraffin. The paraffin blocks were cut at 5 *μ*m using a microtome and deparaffinized tissue slices were subjected to Masson and Hematoxylin and Eosin (H&E) for histological examination. Immunohistochemical was used to investigate the expression of *α*-SMA, Col-I, and TGF-*β*1. In brief, after being deparaffinized, paraffin sections were treated with 1.0% periodate for 1 min to block endogenous peroxidase activity. Then paraffin sections were treated with citrate buffer in a microwave oven for 10 min (antigen retrieval). Sections were washed by phosphate buffered saline (PBS) three times (5 min per time) once cooled to room temperature. After washing, sections were blocked with normal goats serum for 30 min at 37°C, followed with primary antibodies against the *α*-SMA antigen (1 : 600, ab5694, Abcam, Cambridge, UK), Col-I antigen (1 : 700, ab34710, Abcam, Cambridge, UK), and TGF-*β*1 antigen (1 : 50, ab25121, Abcam, Cambridge, UK) overnight at 4°C. Negative control group was made by the remaining slide. Next day, after washing the slides thrice with PBS (5 min per time), sections were incubated with secondary antibody for 10 min at 37°C. PBS washed and sections were treated with streptavidin-biotin-peroxidase complex for 10 min. After washing, diaminobenzidine (DAB) was added as a visualizing agent. Hematoxylin was used for nuclear staining. The sections were examined under light microscope at a photodocumentation facility (Olympus, Tokyo). For each tissue section, the number of positively stained cells was calculated from ten different 400x magnified fields under light microscope.

### 2.6. Content of HYP, Col-I, SOD, MDA, GSH, 8-Iso-Prostaglandin, and T-AOC in Lung Homogenates

The content of HYP in lung homogenate was measured by HYP kits (A030-2, Nanjing Jiancheng Biochemical Institute, Nanjing, China) as our previous study [12–14]. Col-I and 8-iso-prostaglandin were all tested by ELISA kits (Col-I: 41582, 8-iso-prostaglandin: 41874, Yuanye Bioengineering, Shanghai, China). SOD and T-AOC were determined by measuring the absorbance at 550 and 520 nm by using detection kits (SOD: A001-1, T-AOC, A015, Nanjing Jiancheng Biochemical Institute, Nanjing, China). The MDA content was observed by the thiobarbituric acid reactive substances (TBARS) assay in accordance with the manufacturer's instructions (MDA: A003-1, Jiancheng Biochemical Institute, Nanjing, China) and the absorbance was measured at a wavelength of 532 nm.

### 2.7. Western Blotting Analysis

Lung tissue samples were weighed (80–100 mg) on days 7, 14, and 28 and homogenized on ice with ice-cold lysis buffer (P0013C, Beyotime Institute of Biotechnology, China) including protease inhibitor, PMSF (Amresco 0754, Biosharp, USA) for 30 min. After centrifugation (12,000 r/min, 10 min at 4°C), the supernatant was collected, packed, and stored in −80°C. Just before using, the loading buffer was mixed into the supernatant at a ratio of 1 : 4 and cooked in boiling water for 10 min. Proteins in the supernatant were separated by SDS-PAGE on a 12% gel and then transferred to PVDF membranes (no. 20130107054, Millipore, USA). The blotted membranes were blocked with 5% nonfat dry milk (w/v) (No. 2013022311, Guangming, China). After 2 hours, blotted membranes were washed by 0.2% Tween-X100 (TBST) three times (10 min per time). Then blotted membranes were incubated at 4°C overnight with anti-*α*-SMA (1 : 100, ab5694, Abcam, Cambridge, UK), anti-NOX4 (1 : 5000, ab133303, Abcam, Cambridge, UK), and anti-*β*-actin (1 : 5000, ab32572, Abcam, Cambridge, UK) antibodies. Next day PVDF membranes were blocked with 5% nonfat dry milk with Peroxidase Conjugated AffniPure Goat Anti-Rabbit lgG (1 : 15000, ZB-2301, ZSGB-BIO, Beijing, China) for 1 h. After being washed with TBST three times (10 min per time), the blots were visualized with ECL reagent (Thermo Scientific, Rockford, IL, USA).

### 2.8. Statistical Analysis

The data are expressed as mean ± SEM. For all the statistical tests, multiple comparisons were performed by one way analysis of variance (ANOVA) with the Tukey–Kramer or Tamhane's tests by SPSS 17.0. In addition, Graphpad prism 5 software was used for plotting graphs. Values of *p* < 0.05 and *p* < 0.01 were considered to be statistically significant.

## 3. Results

### 3.1. Effect of DBTG on PF in Rats

**Figure 1:**
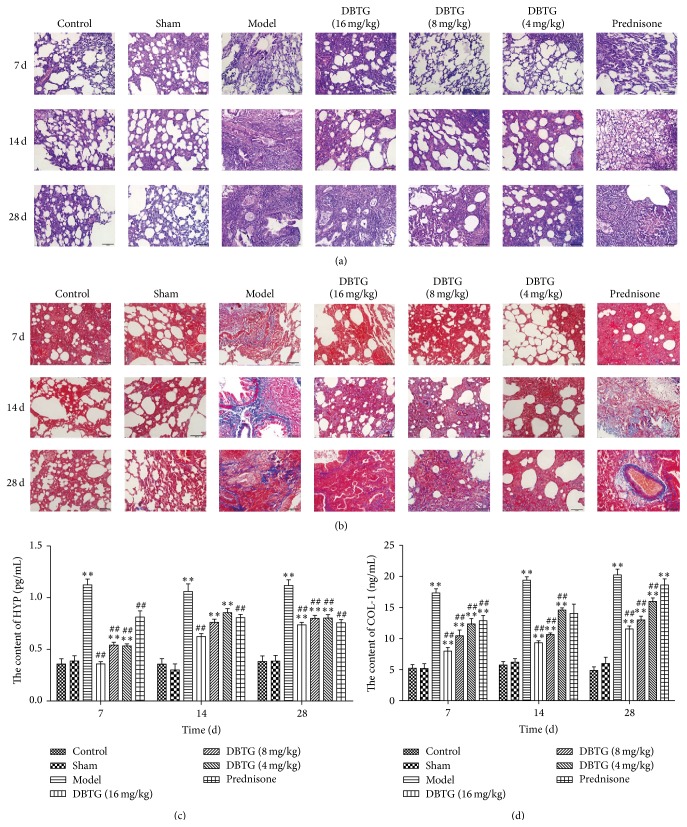
Effect of DBTG on lung fibrosis in BLM-induced rats PF. (a) H&E staining of rat lung tissues (400x); (b) Masson staining of rat lung tissues (400x); (c) content of HYP in lung tissues; (d) content of COL-I in lung homogenates. DBTG could reduce the degree of PF. Data were expressed as mean ± SEM, *n* ≥ 5, ^*∗*^ *p* < 0.05 versus control group, ^*∗∗*^ *p* < 0.01 versus control group; ^#^ *p* < 0.05 versus model group, ^##^ *p* < 0.01 versus model group.

In order to show the role of DBTG on PF, indexes of PF like H&E staining, Masson staining, content of HYP, and Col-I were all tested. Results from H&E staining** (** Figure 1(a)) and Masson staining (Figure 1(b)) indicated that DBTG could reduce the degree of PF compared with the model group. In addition, the content of HYP (Figure 1(c)) and Col-I (Figure 1(d)) of rats with DBTG treatment was lower than the model group significantly (*p* < 0.05 or *p* < 0.01).

### 3.2. Effect of DBTG on Expression of TGF-*β*1 and *α*-SMA

**Figure 2:**
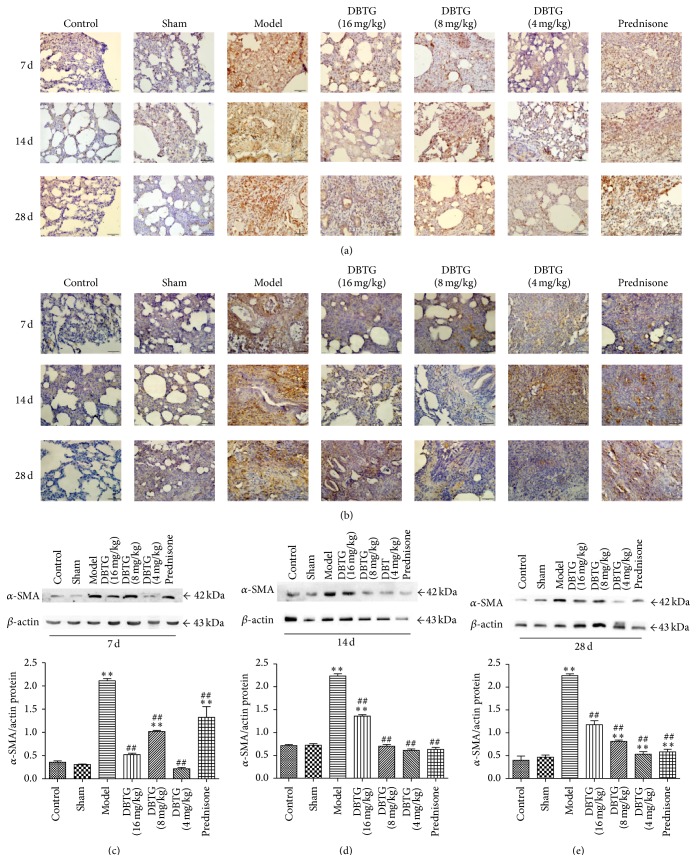
Effect of DBTG on expression of TGF-*β*1 and *α*-SMA in BLM-induced rats PF. (a) Immunohistochemistry of TGF-*β*1 (100x); (b) immunohistochemistry of *α*-SMA (100x); (c) representative western-blots of *α*-SMA, *β*-actin at 7 days (*n* = 3); (d) representative western-blots of *α*-SMA, *β*-actin at 14 days (*n* = 3); (e) representative western-blots of *α*-SMA, *β*-actin at 28 days (*n* = 3). The expression of TGF-*β*1 and *α*-SMA were downregulated by DBTG comparing to model group. Data were expressed as mean ± SEM, *n* ≥ 5, ^*∗*^ *p* < 0.05 versus control group, ^*∗∗*^ *p* < 0.01 versus control group; ^#^ *p* < 0.05 versus model group, ^##^ *p* < 0.01 versus model group.

TGF-*β*1 and its downstream regulatory protein *α*-SMA are important signs of PF. They were detected by immunohistochemistry or western-blot analysis. In Figures 2(a) and 2(b), the expression of TGF-*β*1 and *α*-SMA could be inhibited in groups that were treated with DBTG by immunohistochemistry testing. At the same time, the expression of *α*-SMA was tested by western-blot analysisat each time point (Figures 2(c), 2(d), and 2(e)). After modeling, *α*-SMA was obviously increased compared to control and sham group (*p* < 0.05 or *p* < 0.01). Treated with DBTG, the expression of *α*-SMA was much lower than the model group on days 7, 14, and 28 (*p* < 0.05 or *p* < 0.01).

### 3.3. Effect of DBTG on the Level of Oxidative Stress in Rats with PF

**Figure 3:**
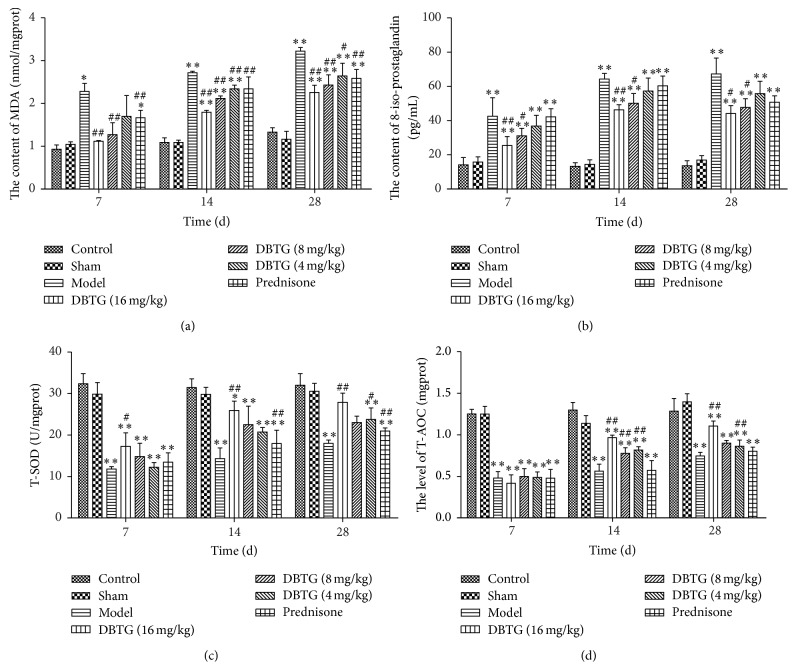
Effect of DBTG on the level of oxidative stress in BLM-induced rats PF. (a) Content of MDA in lung homogenates; (b) content of 8-iso-prostagland in lung homogenates; (c) content of T-SOD in lung homogenates; (d) level of T-AOC in lung homogenates. Oxidative stress indicators like MDA and 8-iso-prostaglandin were reduced and antioxidant indicators such as T-SOD and T-AOC were increased by treatment of DBTG. Data were expressed as mean ± SEM, *n* ≥ 5, ^*∗*^ *p* < 0.05 versus control group, ^*∗∗*^ *p* < 0.01 versus control group; ^#^ *p* < 0.05 versus model group, ^##^ *p* < 0.01 versus model group.

The levels of MDA, 8-iso-prostaglandin, SOD, and T-AOC (Figure 3) were detected to observe the degree of oxidative stress in lung tissue after modeling. Results from Figure 3 showed that the balance of oxidative stress was broken in PF rats compared with the control and sham group (*p* < 0.05). Apart from this, the rats treated with DBTG demonstrated the reduced oxidative stress indicators like MDA (Figure 3(a)), 8-iso-prostaglandin (Figure 3(b)), and the upregulated antioxidant indicators such as T-SOD (Figure 3(c)) and T-AOC (Figure 3(d)) in contrast with the model group (*p* < 0.05 or *p* < 0.01).

### 3.4. Effect of DBTG on Expression of NOX4

**Figure 4:**
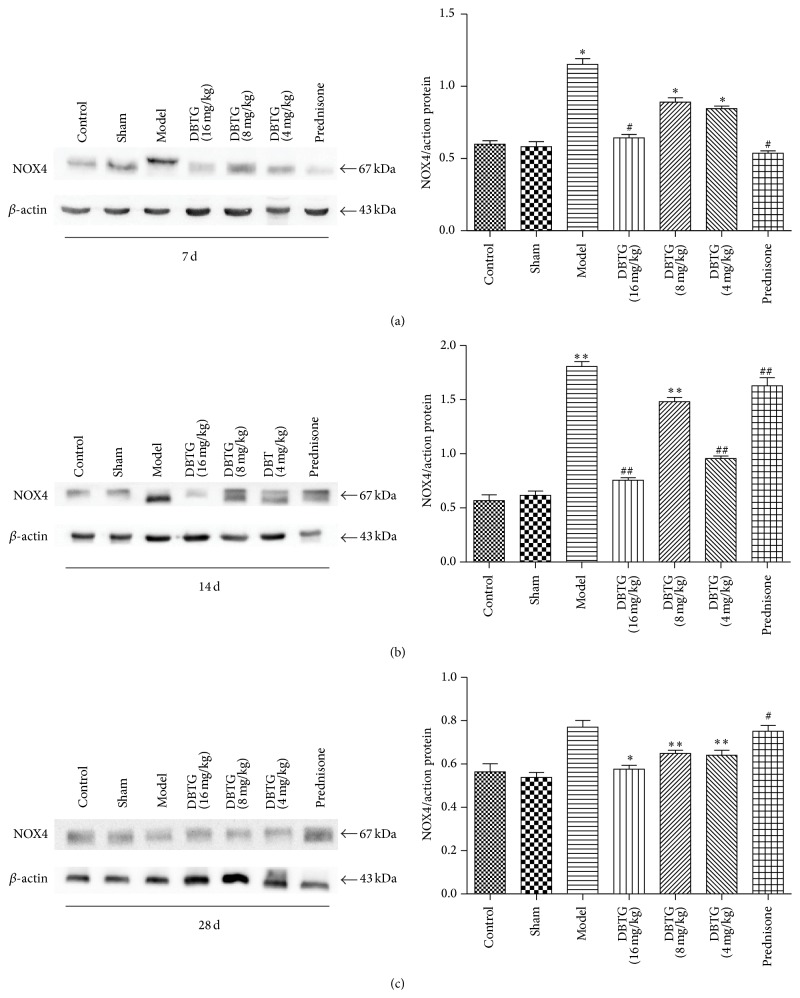
Effect of DBTG on expression of NOX4 in BLM-induced rats PF. (a) Representative western-blots of NOX4 and *β*-actin at 7 days (*n* = 3); (b) representative western-blots of NOX4 and *β*-actin at 14 days (*n* = 3); (c) representative western-blots of NOX4 and *β*-actin at 28 days (*n* = 3). The expression of NOX4 was inhibited in DBTG treatment groups contrast with model group. Data were expressed as mean ± SEM, ^*∗*^ *p* < 0.05 versus control group, ^*∗∗*^ *p* < 0.01 versus control group; ^#^ *p* < 0.05 versus model group, ^##^ *p* < 0.01 versus model group.

NOX4 plays a critical role in oxidative stress. To investigate the effect of DBTG on NOX4, western-blot analysis was performed (Figure 4). The expression of NOX4 was significantly decreased especially on days 7 and 14 after BLM modeling compared with control and sham group. (*p* < 0.05 or *p* < 0.01). These results indicated that DBTG could inhibit NOX4.

## 4. Discussion

PF is a serious progressive chronic lung disease that is incurable in current treatment and usually results in death [16]. Until now, the pathogenesis of PF remains unknown. So far, there is no effective drug for the treatment of PF. Since the current treatments of PF like anti-inflammatory or anti-immune are ineffective, so drugs with curative and low toxicity are urgently needed.

Before researching the effective antifibrotic medicine, pathogenesis of PF should be understood clearly. At present, the mechanism of PF is unclear and there are mainly several different theories [17–19]. In spite of these theories, TGF-*β*1 is currently recognized as one of the most important profibrotic growth factors in the development of PF [1]. Besides that, studies have also confirmed that inflammation factors such as Th1/Th2, IL7, and NF-*κ*B contributed to the process of PF [20–22]. With the deepening research of oxidative stress, recent evidences have showed that oxidative stress plays an important role in PF [23, 24]. When body is subjected to external stimuli, oxidative stress will be induced. Because when oxidative stress happened, reactive oxygen species (ROS) and antioxidants were imbalanced and excessive ROS could damage the normal, physiological function of proteins, lipids, nucleic acids, and other macromolecules in many different cells directly or indirectly. NOX4 belongs to the NADPH oxidase (NOX) family of enzymes that catalyze the reduction of O_2_ to form ROS and it might promote the progress of PF by increasing differentiation of myofibroblast [5].

DBTG is one of the active components isolated from DBT. Our previous studies suggested that DBTG attenuated BLM-induced PF in rats via inhibition of ECM [12, 13]. Since the relationship between DBTG and oxidative stress is unclear in PF, in this study, we explored whether DBTG affects oxidative stress via suppressing NOX4 to inhibit PF. From results of H&E (Figure 1(a)) and Masson staining (Figure 1(b)), the model of PF in rats was successful. Compared with model group, DBTG significantly reduced the content of HYP (Figure 1(c)) and Col-I (Figure 1(d)), especially for the dose of 16 mg·kg^−1^. The results proved that DBTG had the ability to mitigate the degree of PF. TGF-*β*1 is a critical factor in PF and *α*-SMA is a downstream protein of TGF-*β*1. They were tested by immunohistochemical and western-blot (Figure 2). In Figure 2, TGF-*β*1 was inhibited with treatment of DBTG and the expression of *α*-SMA was downregulated.

**Figure 5:**
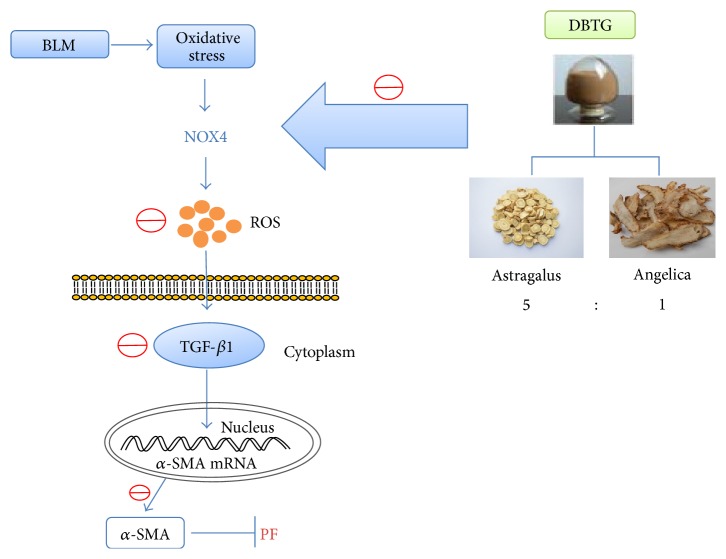
DBTG attenuates BLM-induced rats PF via regulating oxidative stress by inhibiting NOX4. In this study, oxidative stress happened in BLM-induced PF. And then, a lot of ROS was synthesized by NOX4 to promote the expression of TGF-*β*1 and its downstream protein *α*-SMA that increased the degree of PF. DBTG could inhibit NOX4 to reduce ROS and control the level of oxidative stress. As the level of oxidative stress was regulated, the expression of TGF-*β*1 and expression of *α*-SMA were both decreased and, at last, the development of PF could be inhibited.

To analyze the level of oxidative stress, MDA, 8-iso-prostaglandin, SOD, and T-AOC were detected by assay kits (Figure 3). It was confirmed that DBTG could increase the level of antioxidant indexes (SOD, T-AOC) and decrease the level of peroxide indexes (MDA, 8-iso-prostaglandin). DBTG might control the development of PF by regulating the level of oxidative stress. And DBTG with the dose of 16 mg·kg^−1^ was one of the strongest control efforts on regulating the level of oxidative stress. Because NOX4 plays an important role in oxidative stress, it was detected by western-blot at each time point (Figure 4). NOX4 was increased after modeling. This may be related to the stress response in rats themselves. And DBTG might decrease the expression of NOX4. As NOX4 plays an important role in oxidative stress, DBTG might protect against PF via controlling the level of oxidative stress by inhibiting NOX4 (Figure 5).

Based on these results we found that the lowest dose should be more effective than the other two. We analyzed that the reasons might be as follows. First, DBTG is an extract from Chinese medicine that has nonsingle active ingredients like Astragaloside II and Astragaloside IV. Different active ingredients and doses usually play different effects. Second, mechanism of traditional Chinese medicine is multitarget and this will influence the efficiency of treatment. Third, the lower dose may have less toxicity that is helpful to treatment. So the lowest dose of DBTG might be more effective than others.

This study demonstrated that DBTG attenuated BLM-induced pulmonary fibrosis via regulating oxidative stress by inhibiting NOX4. However, the mechanism of DBTG inhibiting NOX4 is still unknown and needs more studies in further future.

## 5. Conclusions

All the above findings suggested that DBTG could attenuate the degree of BLM-induced PF in rats via inhibiting NOX4 to control the level of oxidative stress. NOX4 may be a potential target for inhibiting PF and DBTG may be a promising candidate for the prevention and treatment of PF.

## Abbreviations

AECs: Alveolar epithelial cells
BLM: Bleomycin
Col-I: Type I collagen
DBT: Danggui Buxue Tang
DBTG: Total glucosides of Danggui Buxue Tang
PF: Pulmonary fibrosis
ECL: Electrochemiluminescence
ECM: Extracellular matrix
EMT: Epithelial to mesenchymal transition
ROS: Reactive oxygen species
H&E: Hematoxylin and eosin
HYP: Hydroxyproline
MDA: Malondialdehyde
NOX4: NADPH oxidase- 4
ROS: Reactive oxygen species
Sham: Sham-operated rats
SOD: Superoxide dismutase
T-AOC: Total antioxidant capacity
TGF-1: Transforming growth factor-*β*1
*α*-SMA: Alpha smooth muscle actin.
